# Cobalt Nanoparticles on Plasma-Controlled Nitrogen-Doped Carbon as High-Performance ORR Electrocatalyst for Primary Zn-Air Battery

**DOI:** 10.3390/nano10020223

**Published:** 2020-01-28

**Authors:** Seonghee Kim, Hyun Park, Oi Lun Li

**Affiliations:** 1 School of Materials Science and Engineering, Pusan National University, Busan 46241, Korea; 2 Department of Naval Architecture and Ocean Engineering, Pusan National University, Busan 46241, Korea; hyunpark@pusan.ac.kr

**Keywords:** cobalt nanoparticles, nitrogen-doped carbon, highly durable electrocatalysts, Zn-air battery

## Abstract

Metal–air batteries and fuel cells have attracted much attention as powerful candidates for a renewable energy conversion system for the last few decades. However, the high cost and low durability of platinum-based catalysts used to enhance sluggish oxygen reduction reaction (ORR) at air electrodes prevents its wide application to industry. In this work, we applied a plasma process to synthesize cobalt nanoparticles catalysts on nitrogen-doped carbon support with controllable quaternary-N and amino-N structure. In the electrochemical test, the quaternary-N and amino-N-doped carbon (Q-A)/Co catalyst with dominant quaternary-N and amino-N showed the best onset potential (0.87 V vs. RHE) and highest limiting current density (−6.39 mA/cm^2^). Moreover, Q-A/Co was employed as the air catalyst of a primary zinc–air battery with comparable peak power density to a commercial 20 wt.% Pt/C catalyst with the same loading, as well as a stable galvanostatic discharge at −20 mA/cm^2^ for over 30,000 s. With this result, we proposed the synergetic effect of transitional metal nanoparticles with controllable nitrogen-bonding can improve the catalytic activity of the catalyst, which provides a new strategy to develop a Pt-free ORR electrocatalyst.

## 1. Introduction

Eco-friendly energy devices such as metal-to-air batteries and fuel cells receive much attention, while the importance of air electrodes for oxygen reduction reactions is increasingly prominent. Mainly, air electrodes often require platinum catalysts to improve their slow oxygen reduction reaction (ORR). However, many studies are attempting to reduce the Pt content or entirely replace Pt due to its low durability, high price, and rarity. Among them, transition metal-based electrocatalysts have exhibited promising ORR performance in basic electrolytes and active research is being done in using these as Pt catalyst replacements [1,2,3,4,5]. Among the various transition metals, cobalt is the most widely used as an alternative catalyst material due to its decent oxygen reduction reaction activity, higher four-electron selectivity, high durability, and low price [6,7,8,9]. On the other hand, cobalt nanoparticles as single active sites supported on a pristine carbon matrix often exhibit low activity compared with platinum catalysts [10,11]. Thus, there are many attempting to improve the intrinsic activity of the catalysts. One of the most typical methods is to synthesize a catalyst using a heteroatom-doped carbon matrix [12,13]. Among them, nitrogen-doped (N-doped) carbon represents a far better performance compared with pristine carbon support [14,15,16,17,18]. The catalyst often shows relatively high catalytic performance in alkaline electrolytes. In the case of N-doped carbon, nitrogen reduces the charge density of the nearby carbon atoms due to the differences in electronegativity of carbon and nitrogen [19]. Most recently, a few studies reported enhanced catalytic activity when combining cobalt nanoparticles with N-doped carbon support, and some of their ORR activity might even be comparable to that of platinum-based catalysts [20,21,22,23,24].

To date, there are different arguments about how various types of nitrogen functional groups affect the activity of catalysts [25,26,27,28,29,30,31,32,33,34]. Chatterjee et al. reported nitrogen-carbon nano-ions with pyridinic-N and pyrrolic-N as dominant catalysts. The catalysts contained a nitrogen content of up to 7.5%, and the author suggested that pyridinic-N worked as an active site for oxygen reduction with notably high activity [31]. On the other hand, Wang et al. found a correlation between the potential cycle and the diminishing concentration of quaternary-N in the catalyst. Based on the density functional theory (DFT) calculation and experimental results, quaternary-N displayed lower Gibbs free energy on the rate-liming step in the ORR reaction compared with pyridinic-N or pyrrolic-N. The author claimed that quaternary-N was responsible for the ORR activity in N-doped carbon [35]. Not only for single controlled C-N bonding, Yan also reported the synergetic effect of quaternary-N- and pyridinic-N-doping on the oxygen reduction reaction by using theoretical calculation [17]. After that, Ning et al. reported the synergetic effect of pyridinic-N and quaternary-N by measuring the transferable electrons of N-doped carbon. They suggested that the kinetic current density of the ORR in alkaline media is depended on the ratio of pyridinic-N and quaternary-N [36]. Additionally, Li et al. reported another synergic effect of amino-N and quaternary-N on ORR, where the experimental results showed that dominant amino-N-doped carbon indicated a higher onset potential, and the incorporation of quaternary-N into amino-N improved the 4-electron reaction selectivity and limiting current density [28].

Recently, a great deal of research has been reported on the development of a heterogeneous atomic-doped carbon catalyst through bottom-up synthesis using heterocyclic compounds through liquid plasma engineering. The approach has many advantages, such as being conducted at room temperature and being able to synthesize a heterogeneous atomic-doped carbon catalyst simply by selecting different precursors [37,38,39,40,41,42,43,44]. On the other hand, due to the low thermal stability of amino-N, it is hard to retain amino-N on the carbon matrix by the conventional synthesis route [27]. Thus, we applied a low-temperature facile plasma synthesizing method to fabricate amino-N and quaternary-N selectively on carbon support by careful selection of the precursors. Although a few studies have reported on metal-free tunable N-doped carbon electrocatalysts and/or metal nanoparticles doped on N-doped carbon, the synergic effect of transitional metal nanoparticles with precisely tunable amino-N and quaternary-N has rarely been reported.

In this study, we fabricated cobalt nanoparticles on a nitrogen-doped carbon catalyst through two heterocyclic compounds, quinoline and aniline, via plasma engineering. A pair of cobalt electrodes were applied as the precursor of metal nanoparticles, while quinoline and aniline were the sources of quaternary-N and amino-N, respectively, in the N-doped carbon support. Through XPS results, we can confirm that quaternary-N and amino-N are successfully retained within the carbon matrix from their corresponding precursors. Further, in the electrochemical performance test, cobalt nanoparticles supported on the mixture of quaternary-N and amino-N-doped carbon ((Q-A)/Co) had higher ORR onset potential and limiting current density than those of a single amino-N-doped carbon (A/Co) or quaternary-N-doped carbon (Q/Co) catalyst.

## 2. Materials and Methods

### 2.1. Synthesis of Co-N/C Catalyst by a Plasma Process

**Figure 1:**
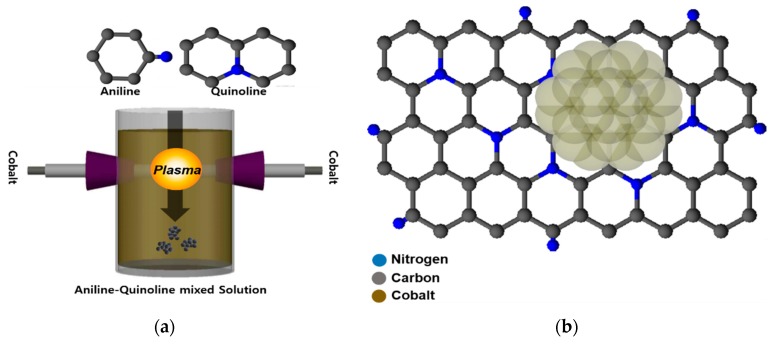
(**a**) Schematic illustration of the solution plasma process and synthesis of the cobalt nanoparticles/N-doped carbon (Co-N/C) catalyst. (**b**) Structure of the N-doped carbon catalyst.

Plasma synthesis was performed between a pair of high purity transition cobalt electrodes (99.999%, Nilaco Co., Ltd., Tokyo, Japan, diameter of 1 mm) inside an organic solution of aniline (A-Co), quinolone (Q-Co), and aniline-quinoline 1:1: mixed solution (Quinoline, Aniline, >99%, Junsei Chemical Co., Ltd., Tokyo, Japan) using a bipolar pulse power supply (MPP-HV02, KURITA, Kyoto, Japan). The plasma was discharged at a voltage of ~4 kV, a frequency of 50 kHz, and a pulse width of 1.0 μs. Stable plasma was discharged for 30 min to obtain cobalt nanoparticles/N-doped carbon (Co-N/C). The solution was filtered using a ϕ 55 mm polytetrafluoroethylene filter, and the resulting filtered carbon powder samples were dried in an oven for 10 h at 80 °C, then heated at 700 °C for 1 h with 1.0 cc/min nitrogen atmosphere to improve their electrical conductivity. The schematic of the plasma synthesis is illustrated in Figure 1.

### 2.2. Structure and Chemical Composition Analysis

The nitrogen absorption-desorption method (BET, Brunauer Emmett Teller; Shimadzu, TriStar-II 3020, Tokyo, Japan) was used for analyzing the surface area, pore volume, and pore diameter of the various Co-N/C catalysts. For morphology and chemical composition analysis, the synthesized carbon samples were characterized using scanning electron microscopy (SEM; JEOL, JSM-7100F, Tokyo, Japan), X-ray diffraction (XRD; Rigaku, Ultima IV, Tokyo, Japan), and X-ray photoelectron spectroscopy (XPS; JEOL, JPS-9010MC, Tokyo, Japan).

### 2.3. Electrochemical Measurements

An electrochemical analyzer (Biologic, VSP, Grenoble, France) was used to analyze the ORR electrochemical properties of the synthesized cobalt-nitrogen-doped carbon. The catalyst ink for the electrochemical analysis was made by adding 4 mg of well-ground Co-N/C-doped carbon catalysts into a mixture composed of 480 μL of distilled water, 480 μL of ethanol, and 40 μL of Nafion^®^117 Solution that was then ultrasonicated for 30 min. A total of 800 μg/cm^2^ of well-dispersed catalyst was applied on a well-polished glass carbon (GC) disk (diameter: 4 mm) electrode (working electrode), where a platinum coil and Hg/HgO (1 M NaOH) were used as the counter and reference electrodes, respectively. After the three-electrode cell was prepared, ORR activity was measured by linear sweep voltammetry (LSV) in O_2_ saturated 0.1 M KOH with a scan rate of 5 mV/s and rotating speed of 1600 rpm, between a potential range of 0.2 to 1.2 V vs. RHE. Chronoamperometry (CA) was conducted at 0.6 V vs. RHE for 30,000 s. In order to investigate the cycle durability of the synthesized catalysts, cyclic voltammetry (CV) was conducted at 50 mV/s for 3000 cycles between a potential range of 0.4 and 1.0 V vs. RHE. The kinetic current of the prepared electrocatalyst was calculated by a rotation rind disk electrode (RRDE). Where i_d_ is disk current, i_r_ is ring current, and C_e_ is collection efficiency (0.42) [45]:

(1) $$\mathrm{Electron}\;\mathrm{transfer}\;\mathrm{number}(n)\;=\;4*(id/\left(id+\left(\frac{ir}{Ce}\right)\right)$$

(2) $$H_{2}O_{2}\;\mathrm{yielding}(\%)\;=\;2*\left(\frac{\frac{ir}{Ce}}{Id+\left(\frac{ir}{Ce}\right)}\right)*100$$

### 2.4. Primary Zn–Air Battery Measurement

The home-made Zn–air battery was assembled with the as-prepared catalysts and loaded on a gas diffusion layer electrode, with a Zn foil as the metal electrode, and 6 M KOH + 0.2 M ZnCl_2_ as the electrolyte. For comparison, a benchmark catalyst (20 wt.% Pt/C) was also measured as the oxygen electrocatalyst. The catalyst ink (same as electrochemical ORR measurement) were well-suspended and dropped onto one face of carbon paper, with a mass loading of 1 mg/cm^−2^. The power density was measured at 10 mV/s from OCV to 0.4 and discharge stability of the sample was measured by discharging at −20 mA/cm^2^ for 30,000 s.

## 3. Results

### 3.1. Properties of Co Nanoparticles/N-Doped Carbon Catalyst

**Figure 2:**
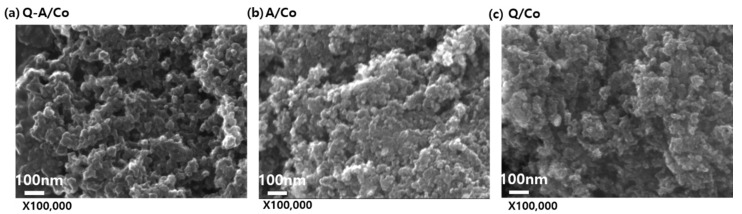
SEM images of the (**a**) quaternary-N and amino-N-doped carbon (Q-A/Co), (**b**) amino-N-doped carbon (A/Co), and (**c**) quaternary-N-doped carbon (Q/Co) catalysts.

**Figure 3:**
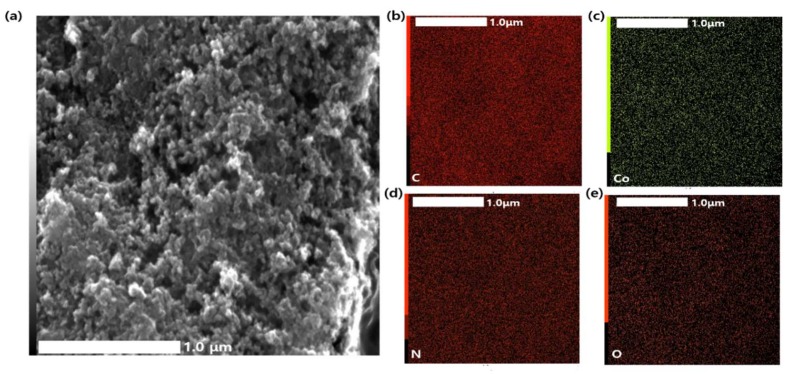
(**a**) SEM image of the Q-A/Co catalyst. (**b**–**e**) EDS mapping of the synthesized Q-A/Co catalyst: (**b**) carbon, (**c**) cobalt, (**d**) nitrogen, (**e**) oxygen.

The SEM images of Q-A/Co, A/Co, and Q/Co are shown in Figure 2a–c. The three electrocatalysts exhibited very similar morphology, and carbon spherical nanoparticles were heavily agglomerated. There were no obvious structural differences from applying various types of nitrogen-carbon precursors during the plasma process. Figure 3 demonstrates the low resolution SEM images with EDS elemental mapping for Q-A/Co, Table S1 summarizes the atomic percentage of each element. It was found that nitrogen from the heterocyclic compound precursors (aniline and quinoline) was successfully retained with a high concentration of around 5% within the carbon matrix. Although the percentage of Co (at.%) was quite small (0.01–0.03%), cobalt nanoparticles formed by electrode sputtering during the plasma process were uniformly deposited on the synthesized carbon matrix (Figure 3c).

**Figure 4:**
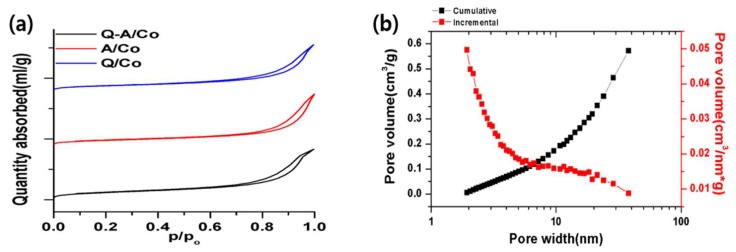
(**a**) BET isotherm linear plot of the three types of synthesized N-doped carbon. (**b**) Barrett-Joyner-Halenda (BJH) adsorption pore distribution of Q-A/Co.

**Figure 5:**
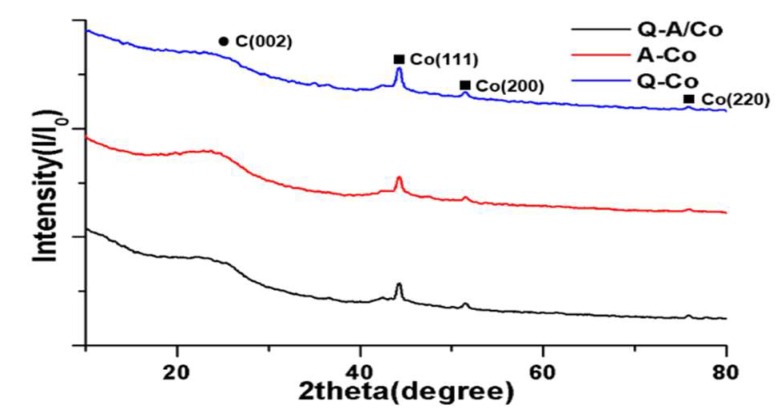
XRD patterns obtained for metal cobalt-N doped carbon.

Figure 4 and Table 1 summarize the porous structure of the synthesized N-doped carbon obtained from the N_2_ adsorption-desorption method. The BET isotherm linear plot in Figure 4a confirms that all synthesized N-doped carbon had a meso-macro porous structure from the hysteresis graph. This is coming from the interparticle voids between the primary carbon particles in the usual solution plasma method synthesized carbon [46]. The BET surface areas of Q-A/Co, A/Co, and Q/Co were 211.1, 210.2, and 206.2 m^2^/g, respectively. This result implies that the porous and structural properties of synthesized Co-N-doped carbon are not affected by the original precursor. In Figure 4b, Q-A/Co exhibits a slightly higher pore volume of 0.67 cm^3^/g and has mainly mesopores with an average diameter of 10.1 nm. To confirm the crystalline structure of the deposited cobalt particle, XRD analysis in Figure 5 clearly indicates pure cobalt metal peaks (Co^0^) at 44°([110], 52°([200]), and 76°([220]). Regardless of the liquid precursor, the composition of the cobalt nanoparticles is identical with similar crystallinity. Combined with the EDS picture and XRD profile, it is clear that the cobalt nanoparticles in their pristine form are successfully fabricated through the plasma process and incorporated into N-doped carbon support.

### 3.2. Chemical Bonding States of Co Nanoparticles/N-Doped Carbon Catalyst

**Figure 6:**
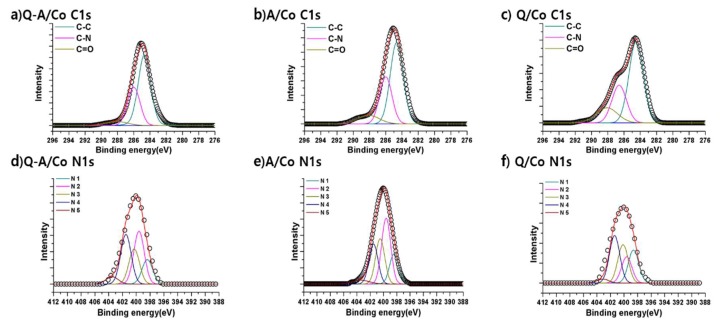
(**a**–**c**) High-resolution C 1s XPS spectra with peak deconvolution of Co-N/C. (**d**–**f**) N 1s XPS spectra with peak deconvolution of Co-N/C.

In order to analyze and understand the effect of nitrogen functional groups on ORR catalytic activity, we applied XPS to figure out the surface nitrogen-carbon bonding states in detail. First, we can confirm that nitrogen is successfully doped into the carbon matrix by the presence of C–N bonding in all synthesized samples from C1s XPS spectra in Figure 6a–c. In order to identify the types of C–N bonding, N1s narrow spectra of Q-A/Co, A/Co, and Q/Co are demonstrated in the corresponding Figure 6d–f. As a reference, the binding energy of amino-N is around 399.4–399.6 eV and quaternary-N is around 401.1–401.5 eV [47]. A/Co, which was synthesized from the aniline precursor, shows a highest amino-N peak of 35%. Additionally, Q/Co fabricated from the quinoline precursor shows a dominant quaternary-N peak of 32%. For Q-A/Co, which was synthesized from a mixed aniline and quinoline solution, two major peaks for quaternary-N (30%) and amino-N (31%) are present equally. From the above XPS results, it is clear that C–N bonding is retained from the heterocyclic compound precursor, and plasma engineering is a facile route to control various types of nitrogen-carbon bonding by choosing the corresponding single or multiple C–N precursors. The relative percentages of nitrogen bonding states of three Co-N-doped catalysts are summarized in Table 2.

### 3.3. Electrochemical Properties of Co Nanoparticles/N-Doped Carbon Catalyst

**Figure 7:**
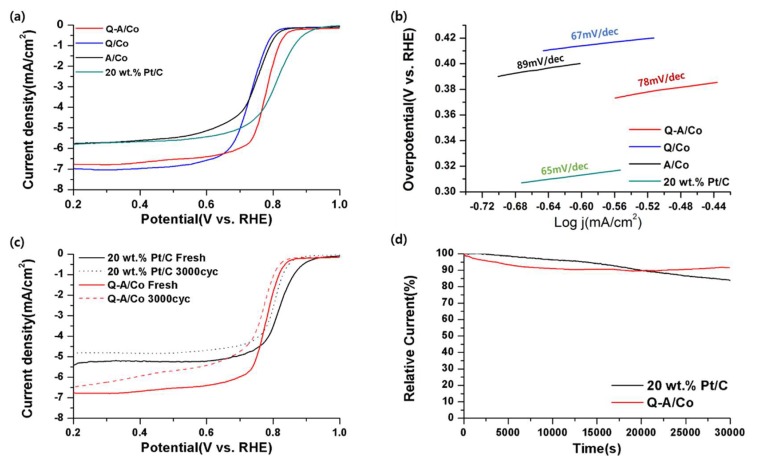
(**a**) Linear sweep voltammetry (LSV) curve of Co-N-doped carbon and 20 wt.% Pt/C at 5 mV/s in O_2_ saturated 0.1 M KOH in rotation speed 1600 rpm. (**b**) Correlated Tafel slope from each LSV curve. (**c**) Cycle durability of Q-A/Co and 20 wt.% Pt/C after 3000 cycles. (**d**) Chronoamperometry (CA) of Q-A/Co and 20 wt.% Pt/C at 0.6 V vs. RHE.

Figure 7a–d shows the electrochemical ORR catalytic activity of synthesized Co-N-doped carbon in 0.1 M KOH. In the polarization curve, A/Co with dominant amino-N demonstrates a slightly higher onset potential (0.85 V vs. RHE) than that of the Q/Co (0.84 V vs. RHE). Figure 7b demonstrates a lower Tafel-slope of Q/Co (67 mV/dec) and Q-A/Co (78 mV/dec) compared with that of A/Co (89 mV/dec), which indicates that quaternary-N doping can improve the ORR reaction kinetics. Figure S1a–c further evaluated the CV curves of each electrocatalyst with a scan rate of 10–80 mV/s, and the double layer capacitance (C*_dl_*) values are derived from each CV based on the equation C*_dl_* = d(Δj)2d(Vb), where V*b* is the scan rate (Figure S1). The electrochemically active surface areas (ECSA) are calculated by ECSA=C*_dl_*/C*s,* of which C_s_ is the specific capacitance of a flat surface with 1 cm^2^ of surface area, and the rugosity can be estimated from ECSA/geometric surface area (0.1256 cm^2^) [48,49]. The calculated C*_dl_* values of A/Co, Q/Co, and Q-A/Co were 14 mF/cm^2^, 14 mF/cm^2^, and 11 mF/cm^2^, respectively. Although ESCA, of all synthesized catalysts, exhibited a similar value of 11–14 mF/cm^2^, the limiting current density of the Q/Co at 0.6 V vs. RHE (−6.41 mA/cm^2^) was much higher than that of the A/Co (−5.17 mA/cm^2^). This result clearly implies that amino-N has a positive effect on the onset potential, whereas quaternary-N improves the reaction kinetics, both of which are similar to previously reported findings [28,50]. Interestingly, Q-A/Co with both dominant amino-N and quaternary-N shows certain differences in ORR activity compared with single amino-N-doped carbon or quaternary-N-doped carbon. The Q-A/Co exhibits better onset potential (0.87 V vs. RHE) and limiting current density (−6.27 mA/cm^2^), which is quite comparable to a commercial 20 wt.% Pt/C catalyst. (0.95 V vs. RHE and −5.43 mA/cm^2^). In reaction selectivity results based on the electron transfer number calculation in Figure S2, Q-A/Co shows the higher 4 e− selectivity of 3.91 compared with those of Q/Co and A/Co (~3.8). The above findings prove the synergetic effect of amino-N and quaternary-N on ORR activities. In the durability test as displayed in Figure 7c,d, the onset ORR potential of Q-A/Co and 20 wt.% Pt/C shift negatively by 10 mV and 20 mV, respectively, after 3000 cycles. Moreover, the relative current density of Q-A/Co reduces by merely 9% while 20 wt.% Pt/C decreases by 16% after 30,000 s at 0.6 V vs. RHE. Both experimental results confirmed that the amino-N and quaternary-N are incorporated into the carbon matrix with high stability. The detailed electrochemical catalytic activity of Co-N-doped carbon and 20wt.% Pt/C are summarized in Table 3.

### 3.4. Primary Zn–Air Battery Test of Co Nanoparticles/N-Doped Carbon Catalyst (Q-A/Co)

**Figure 8:**
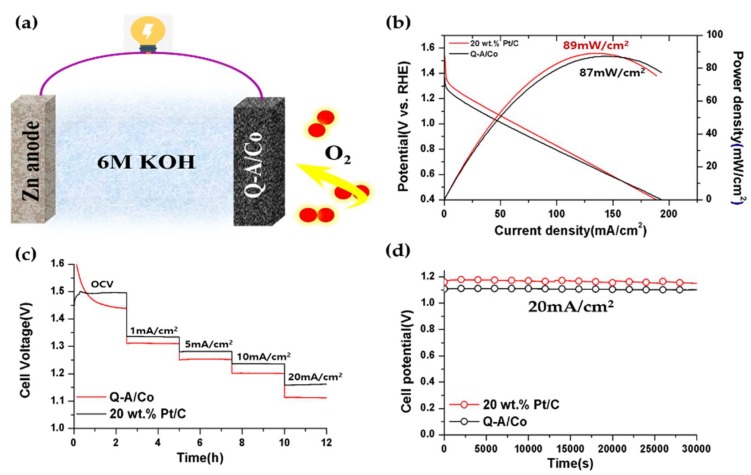
(**a**) Graph for the home-made Zn-air battery. (**b**) Discharge power density of Q-A/Co at 10 mV/s. (**c**) OCV and step voltage of Q-A/Co. (**d**) Discharge stability of Q-A/Co at 20 mA/cm^2^.

Since Q-A/Co outperformed other as-synthesized electrocatalysts, it was further applied as the air cathode in a home-made primary aqueous Zn–Air battery cell, in order to examine its performance in a practical application. In open circuit voltage, the Q-A/Co catalyst exhibited an open-current voltage (OCV) of 1.43 V, which is only slightly lower than that of 20 wt.% Pt/C (1.49 V). On the other hand, the peak power density of Q-A/Co (87 mW/cm^2^) is almost similar to the Pt/C (89 mW/cm^2^) due to the higher discharge current density (Figure 8b). Although the step-voltage discharge of Q-A/Co (Figure 8c) shows lower voltage in the low current density range between 1 and 20 mA/cm^2^, Q-A/Co works comparably to the commercial 20 wt.% Pt/C catalyst in a higher current density region above 50 mA/cm^2^. During the galvanostatic discharge at −20 mA/cm^2^, the Q-A/Co catalyst displays a stable discharge for 30,000 s (Figure 8d). Based on the Zn–air battery test, Q-A/Co is a potential noble-free ORR catalyst to replace expensive noble Pt/C in primary Zn–air batteries with the high peak power density and durable discharge performance.

## 4. Conclusions

Consequently, in this study, cobalt with dominant amino-N- and quaternary-N-doped carbon was synthesized through a plasma process. Both EDS and XRD results indicated that Co particles were deposited on the carbon matrix with nitrogen. From the XPS surface analysis, Q-A/Co exhibited both major amino-N and quaternary-N peaks evenly. This result clearly indicated that aniline and quinoline precursors can be applicable as selective N-C bonding precursors. From the electrocatalytic activity measurement, we confirmed that amino-N had a positive effect on ORR onset potential, whereas quaternary-N improved the limit current density, as reported. In particular, the combination of amino-N and quaternary-N provided a synergetic effect to better the onset potential and limit current density. Q-A/Co displayed a relatively high catalytic activity in terms of onset potential (0.87 V vs. RHE) and limit current density (−6.39 mA/cm^2^), which is only slightly inferior to that of commercial 20 wt.% Pt/C. In a primary Zn–air electrode test, Q-A/Co further exhibited high potential from its high peak power density (87 mW/cm^2^) and durable discharge performance. From the above results, we suggested that plasma engineering is a facile route to fabricate transition metal nanoparticles on selective amino-N and quaternary-N. When combining the synergic effect of transitional metal-based nanoparticles with the controlled N-doped carbon matrix, the noble metal-free catalysts might be further developed as promising candidates to replace the state-of-the-art Pt/C in fuel cell and metal-air batteries.

## Figures and Tables

**Table 1:** Textural parameters of Q/A-Co derived from the N2 adsorption-desorption isotherms.

	BET Surface Area	BJH Adsorption Pore Volume	BJH Adsorption Average Pore Width
Q-A/Co	211.1 m^2^/g	0.6711 cm^3^/g	10.1 nm
A/Co	210.2 m^2^/g	0.6708 cm^3^/g	13.08 nm
Q/Co	206.2 m^2^/g	0.5871 cm^3^/g	15.3 nm

**Table 2:** Nitrogen bonding states of three Co-N-doped catalysts from the deconvolution of N 1s spectra.

Bonding	Binding Energy	Q-A/Co	A-Co	Q-Co
		Relative Percentage (%)		
N1(Pyridinic-N)	398.4–398.6 eV	14	16	22
N2(Amino-N)	399.4–399.6 eV	31	35	18
N3(Pyrrolic-N)	400.1–400.3 eV	20	24	25
N4(Quaternary-N)	401.1–401.5 eV	30	21	32
N5(Oxide-N)	403.3–403.7 eV	5	4	3

**Table 3:** Summary of the electrochemical catalytic activity of Co-N-doped carbon and 20 wt.% Pt/C.

	Q-A/Co	Q/Co	A/Co	20 wt.% Pt/C
Onset Potential	0.87 V vs. RHE	0.84 V vs. RHE	0.85 V vs. RHE	0.95 V vs. RHE
Potential at −3 mA/cm^2^	0.78 V vs. RHE	0.74 V vs. RHE	0.74 V vs. RHE	0.81 V vs. RHE
Current density At 0.6 V vs. RHE	−6.27 mA/cm^2^	−6.41 mA/cm^2^	−5.17 mA/cm^2^	−5.43 mA/cm^2^
Potential at −3 mA/cm^2^ After 3000 cycles Figure 7c	0.77 V vs. RHE	-	-	0.79 V vs. RHE
Current density After 3000 cycles Figure 7d	9% decrease	-	-	16% decrease

## Supplementary Materials

The following are available online at https://www.mdpi.com/2079-4991/10/2/223/s1. Table S1: Atomic percent of A/Co, Q/Co, Q-A/Co from EDS, Figure S1. (a–c) CV curves of synthesized catalysts with scan rate 10–0 mV/s in 0.1M KOH, (d) Current density differences plot with scan rate. ΔJ is current differences between J*a* (anodic current) and J*c* (cathodic current) at potential 0.5 V vs. RHE which is non faradaic region; Figure S2. (a) Electron transfer number and, (b) peroxide yielding of Q-A/Co, A/Co, Q/Co.
