# Supplementary material for: Cobalt Nanoparticles on Plasma-Controlled Nitrogen-Doped Carbon as High-Performance ORR Electrocatalyst for Primary Zn-Air Battery
Source: Nanomaterials (Basel). 2020 Jan 28;10(2):223. doi: 10.3390/nano10020223 (PMC7074963; doi:10.3390/nano10020223)
Supplement: Supplementary file 1 [file nanomaterials-10-00223-s001.pdf]

Supplementary Materials

Table 1. Atomic percent of A/Co, Q/Co, Q-A/Co from EDS.

At. %	C	N	O	Co
Q-A/Co	94.75	5.16	0.05	0.02
Q/Co	94.71	5.17	0.06	0.02
A/Co	94.87	5.07	0.02	0.03

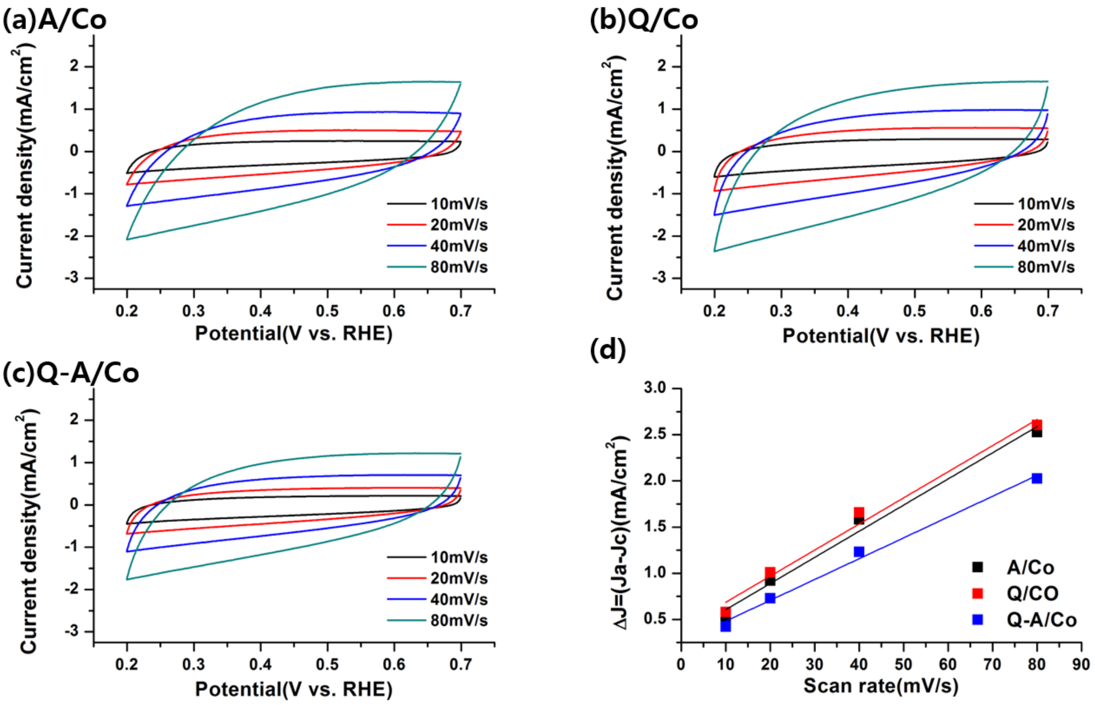

Figure 1. (**a**–**c**) CV curves of synthesized catalysts with scan rate 10–80 mV/s in 0.1 M KOH, (**d**) Current density differences plot with scan rate. ΔJ is current differences between J*a* (anodic current) and J*c* (cathodic current) at potential 0.5 V vs. RHE which is non faradaic region.

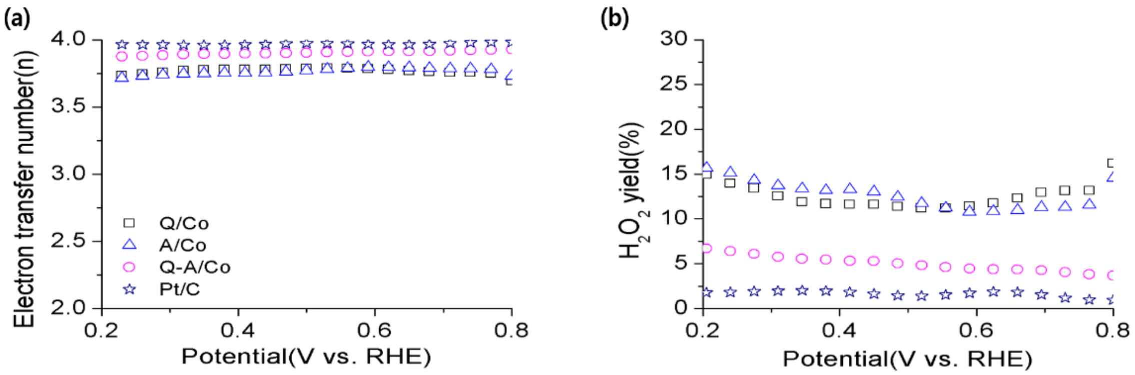

Figure 2. (**a**) Electron transfer number and, (**b**) peroxide yielding of Q-A/Co, A/Co, Q/Co.
